# Feasibility of LMA Supreme for airway management in unconscious patients by ALS paramedics

**DOI:** 10.1186/s13049-015-0105-3

**Published:** 2015-02-26

**Authors:** Sami Länkimäki, Seppo Alahuhta, Tom Silfvast, Jouni Kurola

**Affiliations:** Helsinki Area Helicopter Emergency Medical Service, Helsinki University Central Hospital, FI-00029 HUS Helsinki, Finland; Centre for Prehospital Emergency Care, Länsi-Pohja Healthcare District, Kauppakatu 25, FI-94100 Kemi, Finland; Department of Anaesthesiology, Medical Research Center Oulu, University of Oulu and Oulu University Hospital, Oulu, Finland; Centre for Prehospital Emergency Care, Kuopio University Hospital, PO Box 1777, FI-70210 Kuopio, Finland

**Keywords:** Laryngeal mask, LMA Supreme, LMA-S, Aspiration, Out-of-conscious, Unconscious, Emergency airway management, RSI

## Abstract

**Background:**

Airway management to ensure sufficient gas exchange is of major importance in emergency care. The accepted basic technique is to maintain an open airway and perform artificial ventilation in emergency situations is bag-valve mask (BVM) ventilation with manual airway management without airway adjuncts or with an oropharyngeal tube (OPA) only. Endotracheal intubation (ETI) is often referred to as the golden standard of airway management, but is associated with low success rates and significant insertion-related complications when performed by non-anaesthetists. Supraglottic devices (SADs) are one alternative to ETI in these situations, but there is limited evidence regarding the use of SAD in non-cardiac arrest situations. LMA Supreme (LMA-S) is a new SAD which theoretically has an advantage concerning the risk of aspiration due to an oesophageal inlet gastric tube port.

**Methods:**

Forty paramedics were recruited to participate in the study. Adult (>18 years) patients, unconscious due to medical or traumatic cause with a GCS score corresponding to 3–5 and needed airway management were included in the study. Our aim was to study the feasibility of LMA-S as a primary airway method in unconscious patients by advanced life support (ALS) trained paramedics in prehospital care.

**Results:**

Three regional Emergency Medical Service (EMS) services participated and 21 patients were treated during the survey. The LMA-S was placed correctly on the first attempt in all instances 21/21 (100%), with a median time to first ventilation of 9.8 s. Paramedics evaluated the insertion to be easy in every case 21/21 (100%). Because of air leak later in the patient care, the LMA-S was exchanged to an LT-D in two cases and to ETI in three cases (23.81%) by the paramedics. Regurgitation occurred after insertion two times out of 21 (9.52%) and in one of these cases (4.76%), paramedics reported regurgitation inside the LMA-S.

**Conclusion:**

We conclude that the LMA-S seems to be relatively easy and quick to insert in unconscious patients by paramedics. However, we found out that there were ventilation related problems with the LMA-S. Further studies are warranted.

## Implications statement

Adequate airway management is of major importance during unconsciousness. In this study we observed that ALS paramedics can use the LMA-S as a primary airway method with a good success rate and short insertion time. We also found out that there were critical problems with the ventilation because of air leak. In our opinion, LMA-S still provides an alternative technique for emergency airway management.

## Introduction

Ensuring an adequate gas-exchange is a cornerstone in the treatment of critically ill patients. Oxygenation and ventilation can be performed without advanced techniques using bag-valve-mask ventilation (BVM) and an oropharyngeal tube (OPA), but BVM may be difficult, even for skilled anaesthetists [1-6]. Advanced airway techniques refer especially to endotracheal intubation (ETI), which is considered to be the gold standard in airway management. The technique is associated, however, with low success rates when performed by non-anaesthetist emergency personnel [7-9].

Emergency medical service (EMS) systems in Finland are tiered. The first tier is a voluntary based first responder unit. The second stage consists of emergency medical technicians (EMT) with basic airway management skills. The third stage is advanced life support (ALS) paramedics with advanced training for airway management. Paramedics use benzodiazepine-based sedatives and short acting opioids for procedural sedation, but neuromuscular blocking agents (NMBA) are not used. The fourth stage is a prehospital emergency physician-staffed unit (HEMS or ground). When advanced airway management is needed, ETI with RSI performed by a prehospital emergency physician is standard prehospital care in Finland. Due to the large rural geography and long distances, however, prehospital emergency physicians are not available for all missions which require advanced airway management. On the other hand, the need for ETI is low [10,11] and as such, it is difficult for an ALS paramedic to gain sufficient experience with advanced airway management to become a skilled EMS provider [12]. The Scandinavian Society of Anaesthesiology and Intensive Care Medicine (SSAI) guideline for prehospital airway management recommends that ETI only be performed by anaesthetists [13], while other emergency care providers should use alternative airway devices, such as supraglottic airway devices (SAD). The use of SAD in prehospital care has previously been studied mainly with OHCA (out-of-hospital cardiac arrest) patients [14,15]. Bosch J et al. used LMA-S also as primary or rescue method with unconscious patients with same kind of results as ours [16]. In our study, LMA-S was a primary airway method to replace ETI. Diggs LA et al. gathered every airway management interventions retrospectively in 2012 in United States. LMA was used in 2911 cases and the success rate was reported to be only 66% [17]. Regurgitation occurred in 8.0% of the all cases and it included also regurgitations as a complication from ETI.

## Objective

The aim of the present study was to assess the feasibility of the LMA-S as the primary method of securing the airway in adult unconscious patients with a Glasgow Coma Scale (GCS) 3–5 when used by ALS paramedics. Our hypothesis was that the LMA-S is easy to insert in unconscious patients by ALS paramedics.

## Methods

The study was a prospective, non-controlled feasibility study conducted in 2009–2010. It was approved by the Institutional Ethics Committee (Kuopio University Hospital, Kuopio, Finland).

The LMA Supreme (LMA-S) is one of the latest SAD variants [18]. It is a single-use airway device with an oesophageal inlet with a moulded cuff tip. The inlet allows the passage of an up to 14 F gastric tube when using LMA-S sizes 4–5. It has an anatomically shaped elliptical semi-rigid stem with integral bite block. Inside, the inflatable cuff has moulded fins to prevent epiglottic obstruction. Because of the oesophageal inlet, there may be advantages over older SADs to reduce the risk of aspiration, but there are no relevant studies published. The LMA-S has been compared to other supraglottic airway devices [19-22] and BVM [23]. It has been used in anaesthetized patients and there is evidence for its efficacy and safety [24,25]. Previous studies have also shown the ease of use by novices in manikins and patients [26-28], as well as in prehospital care [16].

Forty paramedics from three ALS-EMS units were recruited to participate in the study. Two units were based in Eastern Finland and one in Western Finland. Areas selected in this study did not have operative prehospital physician to participate the missions. Before the introduction of LMA-S, paramedics usually intubated these patients with medications used also in this study. Decision for airway management on-scene in Western Finland was made by physician in local health care centre or in local hospital by phone, and in Eastern Finland by emergency physician. The level of expertise in ETI was low in these areas and neither standardized training nor local Standard Operating Procedure (SOP) for airway management was available in those days, only national guideline.

The paramedics underwent a one-hour lecture and hands-on manikin training session (Ambu Airway Man®, Ambu A/S, Denmark) before they participated in the study. An emergency physician evaluated the training insertion with an LMA-S size 4 and required two successful performances. Disposable LMA-S (The LMA ® Company, England) sizes 4 and 5 were used.

Adult (>18 years) patients who were unconscious due to medical or traumatic cause with a GCS score corresponding to 3–5 and needed a secured airway (low oxygen saturation, need for neuroprotection) were included in the study. According to national guidelines for prehospital airway management on the time of the study, the GCS below 8 was sufficient indication for paramedic airway management. Paramedics assessed the patient’s GCS after arriving at the scene and contacted the region’s prehospital emergency physician on-call, who made the decision for including the patient in the study according to the written study protocol. Exclusion criteria were out-of-hospital cardiac arrest (OHCA), severe facial trauma, age less than 18 years, and the presence of a prehospital emergency physician on scene.

Before the insertion of the LMA Supreme, the patients were preoxygenated for two minutes with an oxygen reservoir mask or BVM. Procedural sedation was administered with alfentanil and diazepam or midazolam. The weight of the patients and the haemodynamic state was estimated and medications were given with precalculated dosages (alfentanil 0.01 mg/kg, diazepam 0.1 mg/kg, midazolam 0.05 mg/kg) according to the consent of emergency physician. During the procedure, the patients’ heart rate, oxygen saturation and blood pressure were measured. After insertion and cuff inflation, the patients were ventilated using a reservoir bag with supplemental oxygen, capnography was attached and the ease of insertion and adequacy of ventilation were evaluated. The paramedics then inserted the gastric tube and while suctioning or injecting air through the tube assessed the correct placement with epigastric stethoscopy. The patients were then transported to hospital.

The paramedics recorded the following data on a special form based on the Utstein-style template [29]: Size of LMA-S; pre-attempt Glasgow Coma Score; ease of insertion (graded as easy or difficult); number of attempts; time to achieve an airway (opening of the mouth to first ventilation); need for BVM between attempts; adequate ventilation (assessed by vapour in the LMA-S tube, chest movement, symmetric breath sounds, first etCO_2_); sedation administered; vomiting (before or after the LMA-S insertion); aspiration (graded clean, soiling in larynx, crackles in breath sounds, soiling inside the LMA-S); insertion of gastric tube (graded easy or difficult); need to change the device to another technique (Laryngeal Tube, LMA Fastrach, intubation or BVM); place of change of airway device (at the scene or during transport) and complications (mouth opening problem, failure to establish airway, excessive leak, problem with the cuff inflation). Pre- and post-procedure vital signs (blood pressure, oxygen saturation and heart rate) was recorded to the ambulance treatment template and it was not included in the study form due to the Ethics committee statement.

Tabulation of the data was carried out in Microsoft Excel 2010. All data are expressed as medians (range) with IQR if not stated otherwise.

## Results

Twenty-one consecutive patients fulfilled the inclusion criteria. The LMA-S was evaluated to be correctly placed on the first attempt in all patients (21/21, 100%), with a median time to first ventilation of 9.8 s (range of 3 – 20 s). The paramedics evaluated the insertion to be easy in all cases. The ventilation was reported to be adequate in almost all patients (19/21, 90.5%). In the remaining two patients, information was not provided. The median EtCO_2_ values at the first ventilation were 5.0 kPa, with a range of 1.1 – 8.90 kPa. In four cases, the first EtCO_2_ value was not recorded on the form. There were no insertion failures, but because of excessive leak later in the treatment period, the LMA-S was exchanged to an LT-D in two cases and to ETI in three cases (23.8%) by the paramedics. In four of these five cases where LMA-S was exchanged to other airway device, clinical signs of aspiration had occurred before the insertion of the LMA-S.

Regurgitation occurred in two patients after insertion (9.5%), and in one of these, the paramedics reported aspirate inside the LMA-S. In ten cases there were signs of aspiration at arriving on the scene (8/10) or before the attempt to place the LMA-S (2/10). The orogastric tube was only placed in nine patients because of the acute nature of the on-scene situation. The orogastric tube was correctly placed 9/21 times, and placement was reported as being easy in eight cases (89%).

Medications prior to airway management were needed in 15 cases. The median dose of drugs administered was alfentanil 0.85 mg, midazolam 5.75 mg and diazepam 6.25 mg.

## Discussion

Our main findings were that ALS-paramedics were able to insert an LMA-S in 100% of the cases on the first attempt, and that the median time of insertion was less than 10 seconds. Secondly, we found that insertion was reported to be easy in all 21 cases. Thirdly, there were problems after insertion with the ventilation in 23.8% of the cases.

The ALS-paramedics were able to successfully insert an LMA-S after a one hour theoretical lecture and hands-on manikin training. The placement was successful on the first attempt in all patients, and adequate ventilation was confirmed in almost every case (90%). Howes et al. [30] reported that with anaesthetised patients, the first-attempt success rate was 86% and the overall insertion success rate was 100% by medical personnel inexperienced in airway management. The difference to the results in our study may be explained by previous airway management skills. Our paramedics had been using ETI and supraglottic airway devices before in their work and it can be assumed that they are more skilled in airway management. Eschertzhuber et al. studied the use of LMA-S by a single anaesthetist and reported the first attempt insertion success rate to be 95% in anaesthetised and paralyzed patients [25]. The median time of insertion was 34 s in that study, compared to 9.8 s in our study. The observed difference may perhaps be partly explained by the critical condition of our patients. In the study of Eschertzhuber et al., the patients were fasted, stable and non-critical, and thus the anaesthetist was not constraint to proceed urgently with securing the airway while in prehospital care the paramedics may have wanted to secure the airway as fast as possible. The difference between the studies might also have been caused by self-reporting bias. In prehospital care it is not possible to have an independent observer on the scene. The paramedics had to perform airway management rapidly because of a possible impaired airway, while the anaesthetist had time for an elective insertion procedure in ASA I-II patients in the operating room. Also, the time from opening the mouth to the first successful ventilation was measured in this study, whereas Eschertzhuber et al. measured the time from picking up the LMA-S to successful insertion. In our study, the paramedics used only sedative medications without the possibility for anaesthetist-level medications, while propofol and opioids according to anaesthetists’ usual practise were used in the study by Howes et al. [30], using non-anaesthetist doctors, medical students, operating department practitioners, anaesthetic nurses and intensive care nurses as participants. Compared to our investigation, our participants were ALS-paramedics with no anaesthetist skills, but with previous airway management skills in emergency situations. Nevertheless, Howes et al. [30] showed that the LMA-S was suitable for use by airway novices after a short training period.

The LMA-S has an oesophageal inlet with a moulded cuff tip. The inlet allows the passage of an up to 14 F gastric tube when using LMA-S sizes 4–5. Because of the oesophageal inlet, there may be advantages over older SADs (for example LMA Fastrach or LT-D) to reduce the risk of aspiration. In this study, paramedics inserted an orogastric tube in nine cases and the insertion was reported as being easy in eight cases (89%). In one case, paramedics needed multiple attempts to succeed. Eschertzhuber et al. evaluated the use of the LMA-S and the insertion of a gastric tube by an anaesthetist [25]. In their study, the insertion success rate for a gastric tube was 92% on the first attempt and 100% overall. Compared to our study, they had a single anaesthetist with a higher level of experience with airway management. Anaesthetists will inevitably master the use of the LMA-S and gastric tube insertion much better than do paramedics after a one hour lecture and manikin training.

Sunde G et al. studied the use of LT in OHCA by paramedics [14] and reported a high number of air leak in 17.6% of the cases. They also observed other complications with a different SAD and different patient group. The air leak after insertion was the commonest problem in our study with the LMA-S, which might have occurred because of incorrectly estimated size of LMA-S. In our study, 23.8% of the cases, the LMA-S was changed to another airway device. In most of these cases, there were clinical signs of aspiration before the LMA-S insertion. Only in one case, there were signs of regurgitation inside the LMA-S tube and this situation led to ventilation problems and exchange of airway device. The regurgitation before the insertion of LMA-S might be a reason for the ventilation problems. In these cases, the paramedics reported, that the insertion was easy and at first the patient was successfully ventilated, but they were forced to change the airway device due to excessive air leak. One reason for this might be the non-standardized circumstances in the prehospital care. The patients were in critical condition with potential full stomach, and they needed extensive care and treatments on the scene, before they were transferred to the ambulance. The management of airways was always performed on the scene.

At the time of this study, the paramedics usually intubated these patients with GCS below 8. The Laryngeal Tube (LT) was in use in some areas, but it was permitted to use only in OHCA. The reason for implementing the LMA-S in prehospital care to the unconscious patients who needed the secured airway was to minimize the problems caused by ETI.

Our study has several limitations. Firstly, this was an evaluation of a single supraglottic device with a limited number of patients. Secondly, self-reporting introduces the potential of bias and underestimation of adverse event rate. The paramedics reported the insertion of LMA-S to be easy in all cases, but there still were problems with the ventilation in 23.8% of the cases. This difference might be influenced because of self-reporting. However, in prehospital scene independent observing is difficult to accomplish. We used structured data forms with uniform definitions and the missing data were mainly the first capnography reading, which does not affect the evaluation of the feasibility of LMA-S insertion. Thirdly, our study was not designed to measure relationship between patient outcome and airway management. Finally, legal, environmental and organizational aspects may limit the generalizability of our observations. There might have been selection bias in selecting those EMS services that had a special interest in participating in this study and who may have therefore had paramedics more skilled in performing airway management. The results might also have been different if a larger number of patients or paramedics could have been involved.

Bosch and others evaluated the effectiveness and suitability of LMA-S in prehospital emergency patients treated by paramedics [28]. Successful insertion was reported in all 50 cases. Airway leakage was noted in 14% of cases. Nevertheless, we believe that our study offers addition information. In the study by Busch et al., the EMS providers were of higher practical competence, i.e. nurse paramedics, compared to ours EMS providers. In addition, the paramedics of the present study underwent a one-hour lecture and hands-on manikin training session while the nurse paramedics in the study by Busch et al. got acquainted and got experience with LMA-S use for a period of 8 months before they participated in the study. In most of the cases the indication for the use of the LMA-S was for cardiopulmonary resuscitation and the LMA-S was used as the second option after one to three unsuccessful ETI attempts in 64% of the cases in the study by Busch et al.

## Conclusion

We found that the LMA-S was relatively easy to use in terms of success rate and insertion time in unconscious patients after a short lecture and manikin training by paramedics with previous training in the use of both ETI and SAD. We also found out that there were critical problems with the ventilation because of air leak, at least in a patients that have already regurgitated before the airway management. We consider that LMA-S is still potential alternative for airway management by paramedics, at least in regions absence of prehospital physician. The LMA-S may protect patients from aspiration at certain point, but because of the small number of patients in the present study, the risk for aspiration should be considered to be one aspect for further studies.
